# Cold autoimmune haemolytic anaemia secondary to Epstein Barr virus infection presenting with peripheral gangrene; case report

**DOI:** 10.1186/1477-9560-10-4

**Published:** 2012-04-18

**Authors:** Suneth Karunarathne, Sajitha Weerasinghe, Dumitha Govindapala, Harshini Fernando, Bhaddika Jayaratne

**Affiliations:** 1 National Hospital of Sri Lanka, Regent Street, Colombo, Sri Lanka

**Keywords:** Cold autoimmune haemolytic anaemia, Haemolysis, Gangrene

## Abstract

A sixty year old male presented with dark urine, symptomatic anaemia and peripheral gangrene following cold exposure. Investigations revealed that he had haemolysis and serological evidence of recent Epstein Barr virus infection. Although acrocyanosis is commonly associated with cold agglutinin disease, gangrene is a rare complication. Management of secondary cold agglutinin disease is mainly supportive.

## Background

Idiopathic/primary cold agglutinin disease is caused by monoclonal IgM while secondary cold agglutinin disease can be due to either monoclonal or polyclonal IgM [1]. Treatment of cold agglutinin mediated haemolysis is difficult [2].

Gangrene is a rare presentation in cold agglutinin disease[3] and there are only few published reports in medical literature.

Clinically significant cold agglutinins occur at titers over 1:1000 and react at 28-31C and sometimes at 37C [4]. For cold agglutinins, the antigens are the i antigen and the I antigen, coded by a gene on chromosome 14q [5]. The haemolysis is due to complement fixation in the intravascular compartment. When blood temperature drops below the thermal maximum of the antibody IgM cold agglutinins bind to erythrocytes, causing agglutination and binding of complement C1 complex. C1 esterase in turn activates C4 and C2, generating C3 convertase which binds and splits C3, causing deposition of C3b on the erythrocytes [6]. Upon warming, IgM removes from the cell surface and the agglutinated cells get detached from each other, while C3b remains bound on cell surface. C3b activates C5, forming the membrane attack complex. This results in intravascular cell lysis. Majority of the C3b-coated erythrocytes are destroyed by reticulo-endothelial cells in the liver by C3b receptor mediated phagocytosis [7] which is extravascular.

Monoclonal cold agglutinins are associated with B-cell neoplasms - Waldenstrm macroglobulinemia, lymphoma, chronic lymphoid leukemia and myeloma but can occur in non haematologic neoplasms too [8]. Polyclonal cold aggltinins secondary to viral infections can also cause transient immune mediated haemolysis. We describe a patient presenting with peripheral gangrene, which is an unusual finding in cold autoimmune haemolytic anaemia.

## Case report

Previously well 60years old Sri Lankan Tamil male had dark urine for 3weeks which he ignored attributing it to change in the weather. He later developed purplish discoloration of fingers and toes associated with pain in extremities 10days prior to admission. He was admitted to the local hospital with progressively worsening shortness of breath even on mild exertion for seven days. On admission he had dry black discoloration of fingers and toes suggestive of dry gangrene. He did not recall any recent febrile illness or sore throat. There was no history of skin rashes, arthralgia or oral ulcers. There were no previous similar episodes.

He had never smoked and took alcohol only occasionally. Nor he did take any prescribed or recreational drugs. There was no history of viral hepatitis. He had not received any blood transfusions and nor there was any sexual promiscuity. He was a daily paid manual worker and had 3 children.

On further questioning he mentioned that they were displaced from their home due to floods and unusually cold weather and had to live in a temporary shelter one month prior to presentation. He was involved in carrying their belongings to dry land knee deep in cold rainwater. His urine colour turned dark about one week later.

**Figure 1:**
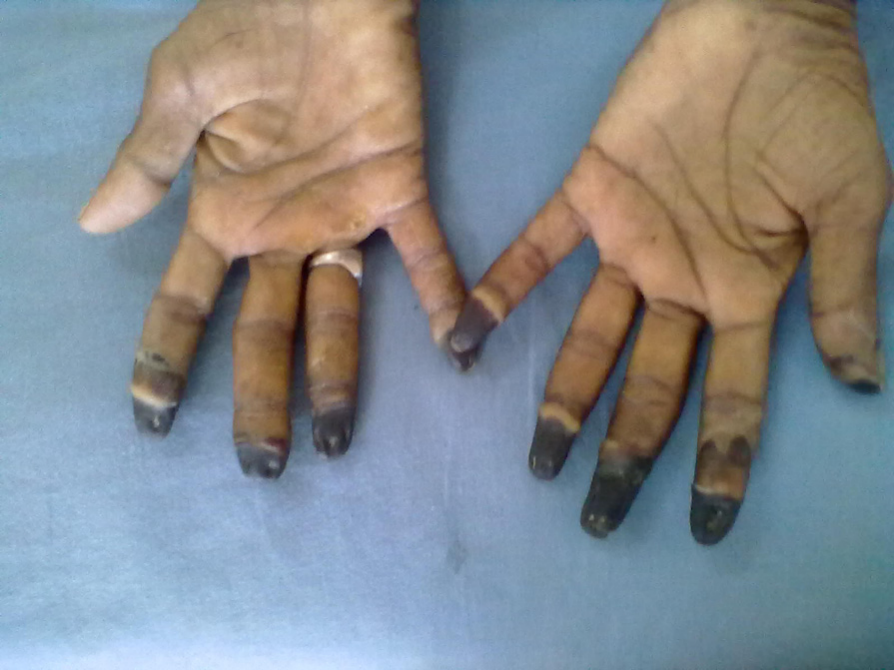
Dry gangrene of fingertips involving nine out of the ten fingers.

**Figure 2:**
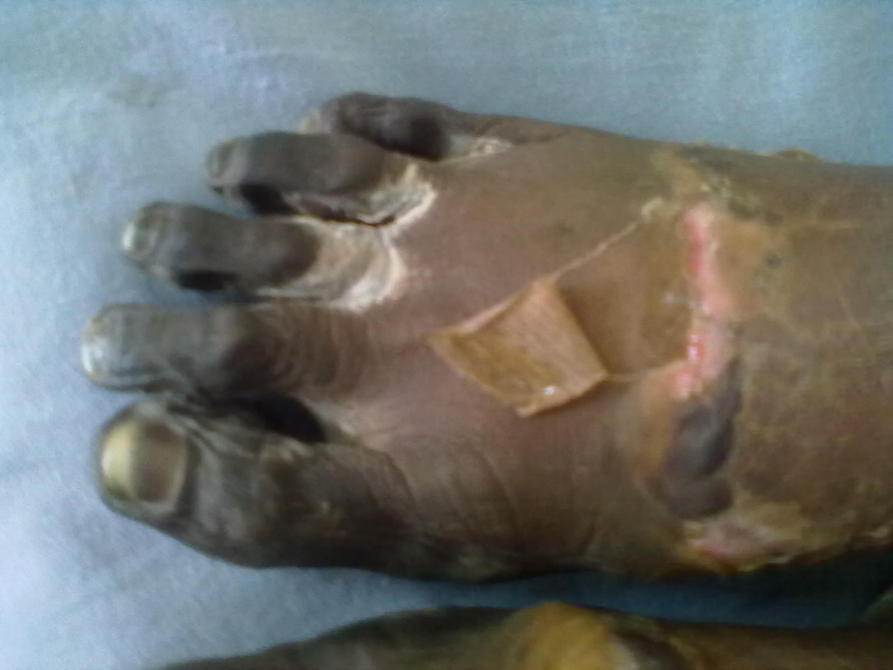
Dry gangrene of toes and forefoot with clear demarcation.

On examination he was febrile and sweaty. He appeared ill but was not in pain. He had severe pallor and was mildly icteric. There was no finger clubbing. Fingers, toes and forefoot were discolored, shrunken and appeared lifeless (Figures1 and 2). Pulse rate was 92bpm, regular with good volume. Jugular venous pressure was not elevated. Blood pressure was 110/70mmHg. On auscultation heart was in dual rhythm with an ejection systolic murmur in aortic area. All peripheral pulses were palpable. Respiratory system examination was normal. No palpable liver, spleen or other masses were detected on abdominal examination. Patient was conscious and rational. No neurological deficits were detected except absent sensation on peripheries which were discoloured.

Laboratory investigations revealed, Hb-2.7g/dl, platelets-432000/l, WBC-15810/l (N 64, L- 19, E- 7, M 9) MCV-106.3fl, MCH-65pg, MCHC-61.1g/dl and reticulocyte count 14%. Total bilirubin 26.8mol/l, Indirect bilirubin 14.6mol/l, Direct bilirubin 12.2mol/l, AST-62u/l, ALT-16u/l, ALP-212u/l,Serum creatinine 89mol/l, Serum sodium 143mmol/l, Serum potassium 3.8mmol/l. Fasting blood sugar 4.7mmol/l erythrocyte sedimentation rate 30mm/Hr. Urine for haemoglobin was positive.

Hepatitis B surface antigen and Hepatitis C antibody were negative. Anti nuclear antibody and HIV serology were also negative. Mycoplasma IgM was negative. Epstein Barr virus IgM was positive with a titre of 1 in 1600. VDRL was non reactive. Blood picture revealed normochromic normocytic red cells, spherocytes, polychromatic cells, nucleated RBC and red cell clumps suggestive of acute haemolysis. White cells showed neutrophil leucocytosis with left shift. Platelets were normal in number with many platelet clumps. Direct antiglobulin test was positive with IgM and anti C3d specificity with strong auto-agglutination at 4C and room temperature and no auto-agglutination at 37C. Cold agglutinin titre was 1 in 2048.

Blood film findings were compatible with severe cold auto-agglutinin mediated peripheral haemolysis with C3d specificity. Serum protein electrophoresis showed polyclonal increase in Gamma globulin region. Cryoglubulins were not detected.

Upper gastrointestinal endoscopy showed multiple small antral ulcers and multiple small duodenal ulcers with no active bleeding.

Vascular surgeon decided no active intervention needed as patient had demarcated dry gangrene. Provisional diagnosis was cold autoimmune haemolysis associated with Epstein Barr virus IgM.

Patient was transfused seven units of O positive red cells over a period of one week. He was started on folic acid 5mg daily. After the blood transfusions he improved symptomatically. Repeat blood film two weeks after admission showed resolution of haemolysis. Patient was advised to avoid cold and to wear gloves and stockings in cold weather. He was discharged home after arranging follow up for occupational therapy. Due to the loss of fingers and toes he was markedly disabled and had to stop working as a labourer.

## Discussion

Polyclonal cold agglutinins are usually acute, transient, and postinfectious, occurring in children and young adults [9]. Infections that can cause cold agglutinin mediated hemolysis include Mycoplasma, Epstein-Barr virus (EBV), cytomegalovirus (CMV), mumps, varicella, rubella, adenovirus, (HIV), influenza and hepatitis C [9].

In the presence of cold agglutinin mediated cell clumping automated cell analysers can give falsely high MCH and MCHC values as they measure red cell clumps as a very dense single cell. This could be avoided by incubation at 37C before analysis [10].

In this case, haemolysis is probably precipitated by polyclonal IgM immunoglobulins secondary to infection with Epstein Barr virus. Recent cold exposure may have precipitated the haemolysis and gangrene due to red cell agglutination. When the red cells reach the peripheries which are cold, IgM antibodies bind to erythrocytes causing red cell clumps, if the clumping is severe enough this may lead to occlusion of microvasculature due to red cell clumps leading to ischemic gangrene.

Gangrene is a rarely described finding in cold autoimmune haemolytic anaemia [3]. When found, it is usually associated with primary cold agglutinin disease [11]. We were unable to find any reports of Epstein- Barr virus associated cold immune haemolysis giving rise to gangrene. Bachmeyer C et al. has described a patient with cold induced immune haemolysis due to T cell lymphoma with necrosis of extremities [3] while a report by Freedman J et al. describes a patient with venous gangrene secondary to mixed cold and warm autoimmune haemolytic anaemia [12]. Patel M et al. have described a patient with Paroxysmal cold haemoglobinuria coexisting with cold agglutinin disease in a patient with syphilis resulting in peripheral gangrene.

This case is clinically informative as it describes a hitherto unreported occurrence of peripheral gangrene associated with Epstein Barr virus associated secondary cold immune haemolysis. Knowledge about this presentation will make clinicians think of this possible albeit rare differential diagnosis when a patient presents with gangrene of extremities.

## Consent

Written informed consent was obtained from the patient for publication of this Case report and accompanying images. A copy of the written consent is available for review by the Editor-in-Chief of this journal.

## Competing interests

The authors declare that they have no competing interests

## Authors contributions

All authors were involved in the management of the patient. SK prepared the manuscript. SW and DG provided valuable input in the literature survey and manuscript preparation. HF and BJ were involved in clinical decision making and they reviewed the manuscript with regard to scientific data. All authors read and approved the final manuscript.

## Authors information

SK and SW are medical registrars attached to the national hospital of Sri Lanka. DG is a senior registrar in general medicine. HF is a consultant physician attached to the National hospital of Sri Lanka while BJ is the consultant hematologist at the same institute.
